# Completion of Treatment for Latent Tuberculosis Infection with Monthly Drug Dispensation Directly through the Tuberculosis Clinic

**DOI:** 10.1371/journal.pone.0048900

**Published:** 2012-11-05

**Authors:** Claudia C. Dobler, Guy B. Marks

**Affiliations:** 1 Respiratory and Environmental Epidemiology, Woolcock Institute of Medical Research and Central Clinical School, University of Sydney, Sydney, Australia; 2 Department of Respiratory Medicine, Liverpool Hospital, Sydney, Australia; BarcelonaUniversity Hospital, Spain

## Abstract

**Setting:**

An Australian metropolitan TB clinic where treatment for latent tuberculosis infection (LTBI) comprises six months of isoniazid, self-administered but dispensed monthly by the clinic.

**Objective:**

To determine the proportion of patients who complete treatment for LTBI and to identify factors associated with non-completion.

**Methods:**

Clinical files of all patients receiving treatment for LTBI between 01/2000 and 12/2010 were reviewed. The study population comprised all patients who were commenced on isoniazid as treatment for LTBI. Odds ratios (OR) for completing treatment were estimated by logistic regression.

**Results:**

Of 216 patients who commenced isoniazid treatment for LTBI, 16 (75%) completed six months treatment. Fifty-three percent of the 53 patients who did not complete treatment dropped out after three months treatment. The mean (SD) age of the patients was 27 (16) years and 123 (57%) were female. The majority of patients (59%) were born overseas and 69% received treatment for LTBI because they were contacts of patients with TB. Patients' sex, age, country of birth, time since immigration for overseas born people, health care worker status, TST conversion status, chest x-ray findings, language, employment status and the indication for which treatment of LTBI was prescribed were not significantly related to treatment completion.

**Conclusion:**

In a setting where isoniazid is dispensed monthly by the TB clinic, a relatively high proportion of patients who commence treatment for LTBI complete the six month scheduled course of treatment. The study did not identify any patient characteristics that predicted treatment completion. Interventions to improve completion rates should extend over the whole duration of treatment.

## Introduction

Treatment of latent tuberculosis infection (LTBI) is a cornerstone of tuberculosis control, especially in settings where tuberculosis (TB) is not endemic [1]. Isoniazid treatment of LTBI has been shown to have an efficacy of approximately 90% in randomized controlled trials in the 1950s and 1960s in which treatment was given for a period of 12 months [1], [2]. A trial conducted by the International Union Against Tuberculosis in Eastern Europe in people with fibrotic pulmonary lesions found that 24 weeks of isoniazid treatment reduced the incidence of TB by 65% and 52 weeks of isoniazid treatment reduced the incidence of TB by 75% [3]. However, 24 weeks treatment prevented more TB cases per case of hepatitis caused. Although no trial has ever evaluated the effectiveness of 9 months of treatment with isoniazid, Comstock recommended 9 to 10 months of isoniazid treatment as optimal based on observations among absconders in the Bethel trial [4]. Today the standard treatment for LTBI in Australia consists of a 6 to 9 months course of isoniazid. In clinical practice the effectiveness of treatment for LTBI is often reduced by poor adherence to therapy, possible attributable to the long duration of therapy, the lack of any perceived disease or symptoms and adverse effects. Thus, overall effectiveness of isoniazid treatment for LTBI measured in randomized controlled trials varied from 25 to 92%, [1], [2] with an overall effect estimate of 60% [5]. This marked gap between efficacy and effectiveness represents a major challenge for program implementation.

The challenge of treatment for LTBI is “convincing the patient of the need for prolonged treatment of a noncontagious infection that may never develop into TB disease, using medications with potential side effects ”[6]. The concept of latent infection is difficult to grasp for many patients, and language problems in foreign born patients often further complicate communication. Previous studies suggest a low proportion of patients who have started treatment for LTBI complete treatment [7]: ranging from 19% (for isoniazid for 9 months) [8] to 82% (for isoniazid for 6 months) [9]. This wide range of estimates implies that factors specific to the setting have a major influence on the adherence.

Socio-demographic and clinical characteristics, health care service design, and the treatment regimen can all have an impact on treatment adherence and thus the proportion of patients who complete treatment. One of the potential barriers to adherence is the need to fill prescriptions and pay for the medications at a dispensary that is separate from the health service provider who recommends and prescribes treatment for LTBI. In our TB clinic we dispense isoniazid free-of-charge monthly directly to patients who are prescribed treatment for LTBI. Patients who do not attend on schedule are contacted by clinic nurses via phone call up to three times. If patients cannot be contacted via telephone, an appointment card is sent to their home address and the treating physician is informed. It is possible that this relatively low intensity intervention is associated with better adherence than has been reported elsewhere.

We aimed to determine the proportion of patients who complete treatment for LTBI in a setting where isoniazid is self-administered for 6 months with monthly drug dispensation through the TB clinic. We further aimed to identify characteristics that were associated with failure to complete treatment as a basis for future targeted adherence interventions.

## Methods

We examined a cohort of individuals who were commenced on self-administered isoniazid as treatment for LTBI between 1^st^ January, 2000 and 31^st^ December, 2010 at the TB clinic of a metropolitan hospital in Sydney, Australia. A list of all potentially eligible patients was obtained from a register at the TB clinic. Data on eligibility and study factors for these patients were obtained from their medical records and the New South Wales Clinical Surveillance System Database and entered into a study database.

### Study factors

We extracted data on sex, age, country of birth, tuberculin skin tests, interferon gamma release assays (IGRA), reason for treatment for LTBI, prescribed treatment regimen, start and end dates of treatment, baseline transaminases, adverse events, termination or interruption of treatment, and reasons for not completing treatment (if applicable).

### Treatment regimen

Isoniazid was prescribed at a daily dose of 300 mg/day for adults and 10 mg/kg/day for children. Patients treated for LTBI with a combination of drugs including isoniazid were not included in this study, as treatment with more than one anti-tuberculous drug was routinely administered via directly observed therapy. Patients were given medication for one month at a time and were instructed to come to the TB clinic to collect another one month supply once they had finished the whole bottle of pills, at which time they were also asked about any adverse events by a TB clinic nurse. It was assumed that all 30 pills in a bottle had been taken (without performing pill count) when the patients presented to collect another one month supply of isoniazid as there were no predetermined drug collection dates. The prescribing physician reviewed the patient 4 weeks after commencement of treatment, and then every 6–8 weeks until completion of treatment. There was some variation in this routine, depending on the perceived risk of or actual experienced adverse events. A supplemental pyridoxine tablet was routinely added to all treatment regimens containing isoniazid for adults and older children. Liver function tests were not performed routinely during follow-up, except when the patient was considered to be at an increased risk of developing drug-induced hepatitis.

### Outcome definitions

Patients who collected the first bottle of isoniazid tablets were considered to have commenced treatment for LTBI. Treatment was defined as completed if six monthly bottles isoniazid were collected by the patient. Patients who were started on isoniazid but were switched to another self-administered drug regimen were considered to have completed treatment if they finished the proposed alternative course of treatment.

TST conversion was defined as an initial TST ≤10 mm with an increase in TST size of ≥6 mm and a TST ≥10 mm at follow up [10]. LTBI was assessed based on the New South Wales Department of Health guidelines on TST interpretation [11]. The term “treatment for LTBI” was used to refer to preventive treatment given to a person considered to be at risk of developing TB, regardless of their TST status and IGRA result.

An adverse event was defined as any change in health status or side effect that was attributed to isoniazid by the treating physician and led to treatment interruption, change or cessation.

### Data analysis

Associations between treatment completion and demographic and clinical characteristics were assessed estimated as odds ratios (ORs) with 95% confidence intervals (95% CIs) using logistic regression. The independent effect of potential predictors of non-completion was estimated using multivariate logistic regression. Missing values were coded as specific subgroup in the multivariate analysis to reduce the risk of selection bias due to excluding subjects with any missing data from the multivariate analysis. We tested the difference in treatment-days between those who completed six months treatment and those who only completed five months treatment using the two samples t-test. Statistical analysis was performed using IBM SPSS Statistics 20 (IBM, Armonk, NY, USA).

### Ethical considerations

The study protocol was approved by the the NSW Population & Health Services Research Ethics Committee.

## Results

### Description of the cohort

**Figure 1:**
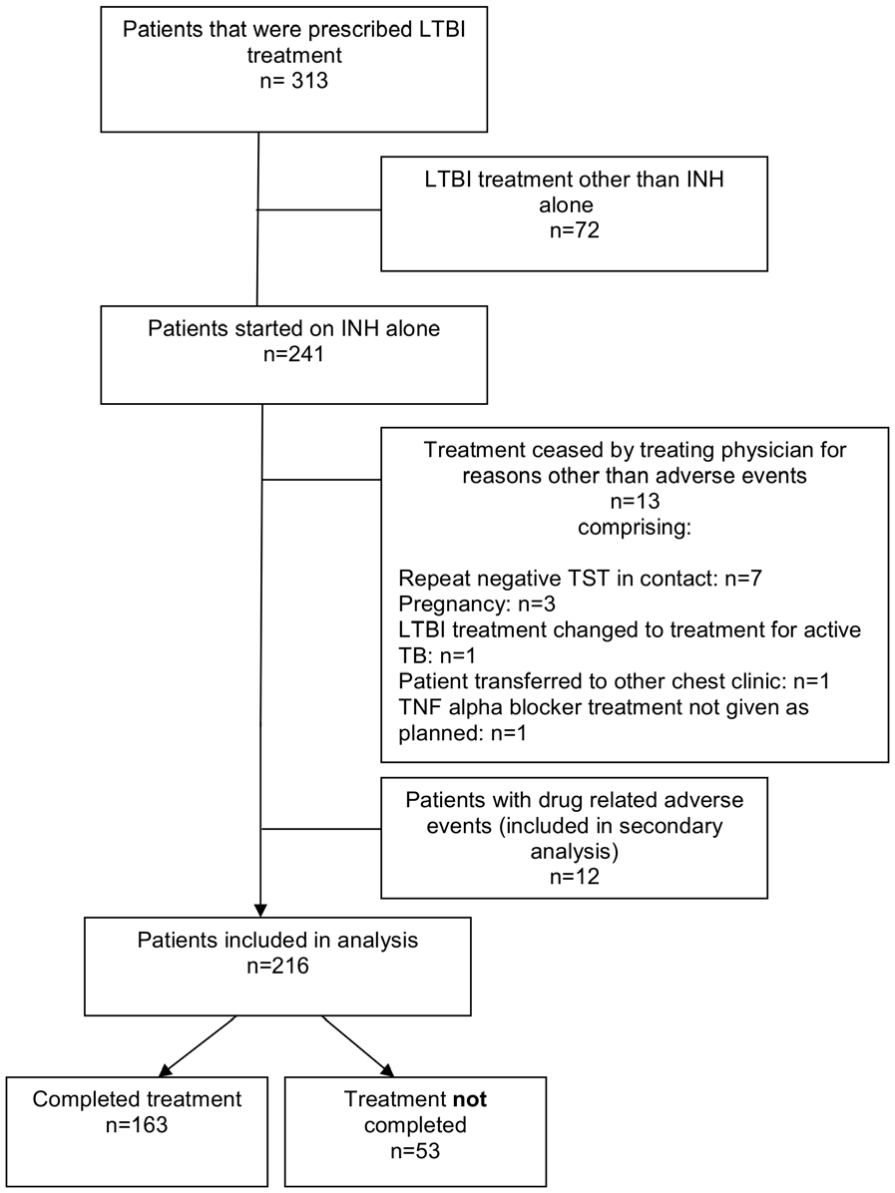
Flow diagram of the study population.

During the study period 241 patients were started on isoniazid treatment for LTBI. Thirteen patients were excluded from the analysis because their treatment was ceased by their treating physician for reasons other than adverse effects of treatment or they were transferred out to another TB clinic and twelve patients were excluded because they had an adverse event, leaving 216 patients for analysis of treatment completion (Figure 1). Six patients had a change of their treatment regimen after they had been started on isoniazid due to isoniazid resistance of *M. tuberculosis* in the index case (n = 3) or drug-related adverse events (n = 3). The HIV status was not routinely assessed among patients with LTBI, and thus HIV results were only available for 6 out of the 216 patients (all negative).

The mean (SD) age of the patients was 27 (16) years and 123 (57%) were female. The majority of patients (59%) were born overseas and 69% received treatment for LTBI because they were contacts of a patient with TB. Other reasons for treatment included planned TNF alpha blocker treatment (n = 40), immunosuppression other than TNF alpha blocker therapy (n = 7), health care worker screening (n = 12), immigration screening (n = 4) and defence force screening (n = 3). Characteristics of the study cohort are shown in Table 1.

**Table 1:** Factors associated with treatment completion

Factor	Level	n	%	n who completed treatment	% completed treatment	Odds Ratio (95% CI) univariate	P value	Odds Ratio (95%CI) multivariate	P value
**Sex**	Female	123	57%	93	76%	1.0 (0.5 to 1.9)	0.95	1.1 (0.6 to 2.2)	
	Male	93	43%	70	75%	1.0		1.0	0.7
**Age**	0–14 years	51	24%	41	80%	1.8 (0.8 to 4.0)		1.3 (0.5 to 3.3)	
	≥35 years	56	26%	46	82%	2.0 (0.9 to 4.4)		1.2 (0.4 to 3.5)	
	15–34 years	109	50%	76	70%	1.0	0.1	1.0	0.9
**Country of birth**	Overseas born, immigration<2 years ago	19	9%	15	79%	1.2 (0.4 to 3.9)		0.8 (0.2 to 3.4)	
	Overseas born, immigration 5–10 years ago	38	18%	28	74%	0.9 (0.4 to 2.1)		0.7 (0.2 ton 1.9)	
	Overseas born, immigration ≥10 years ago	69	32%	51	74%	0.9 (0.4 to 1.8)		0.6 (0.2 to 1.5)	
	Overseas born, no info on date of immigration	2	1%	2	100%	not estimable		not estimable	
	Australian born	88	41%	67	76%	1.0	0.99	1.0	0.8
**Health care worker^a)^**	Yes	21	10%	16	76%	1.0 (0.4 to 3.0)		1.2 (0.3 to 4.6)	
	No	195	90%	147	75%	1.0	0.9	1.0	0.8
**TST conversion^b)^**	yes	58	27%	41	71%	0.7 (0.4 to 1.4)		0.8 (0.4 to 1.8)	
	no	158	73%	122	77%	1.0	0.3	1.0	0.6
**CXR**	abnormal	17	8%	16	94%	5.7 (0.7 to 44.3)		6.1 (0.7 to 51.2)	
	Missing info	2	1%	2	100%	not estimable		not estimable	
	normal	197	91%	145	74%	1.0	0.2	1.0	0.3
**Reason for treatment for LTBI**	TB household contact	92	43%	70	76%	1.0	0.1	1.0	0.2
	TB non-household contact	57	26%	37	65%	0.6 (0.3 to 1.2)		0.5 (0.2 to 1.2)	
	immunosuppression	47	22%	34	85%	1.8 (0.7 to 4.6)		1.9 (0.6 to 6.4)	
	other	20	9			1.3 (0.4 to 4.2)		1.0 (0.2 to 4.9)	
**Patient's language of communication**	Other than English	23	11%	17	74%	0.9 (0.3 to 2.5)		0.8 (0.2 to 2.8)	
	Missing info	3	1%	3	100%	not estimable		not estimable	
	English	190	88%	143	75%	1.0	0.99	1.0	0.9
**Employment status**	Unemployed	8	4%	7	88%	1.3 (0.7 to 2.5)		2.0 (0.2 to 18.6)	
	missing info	9	4%	6	67%	0.7 (0.2 to 3.0)		0.5 (0.1 to 2.7)	
	employed or child or student or other	199	92%	150	75%	1.0	0.6	1.0	0.6

a) Includes all health care workers not just those who received LTBI treatment as a consequence of routine health care worker screening

b) TST conversion defined as an initial TST ≤10 mm with an increase in TST size of ≥6 mm and a TST≥10 mm at follow up

c) Adjusted for all covariates except age and baseline transaminases, as those two variables are likely to be associated with adverse events based on previous clinical experience.

### Treatment completion

Seventy-five per cent (163/216) of the cohort completed treatment. Patients' sex, age, country of birth, time since immigration for overseas-born people, health care worker status, TST conversion status, chest x-ray findings, language spoken at home, employment status and the indication for which treatment of LTBI was prescribed were not significantly related to treatment completion. (Table 1). People with isoniazid-related adverse events were excluded from the main analysis; secondary analysis including these 12 patients showed that they were less likely to complete treatment than those without any adverse events (adjusted OR 0.20, 95% CI 0.05 to 0.86).

**Figure 2:**
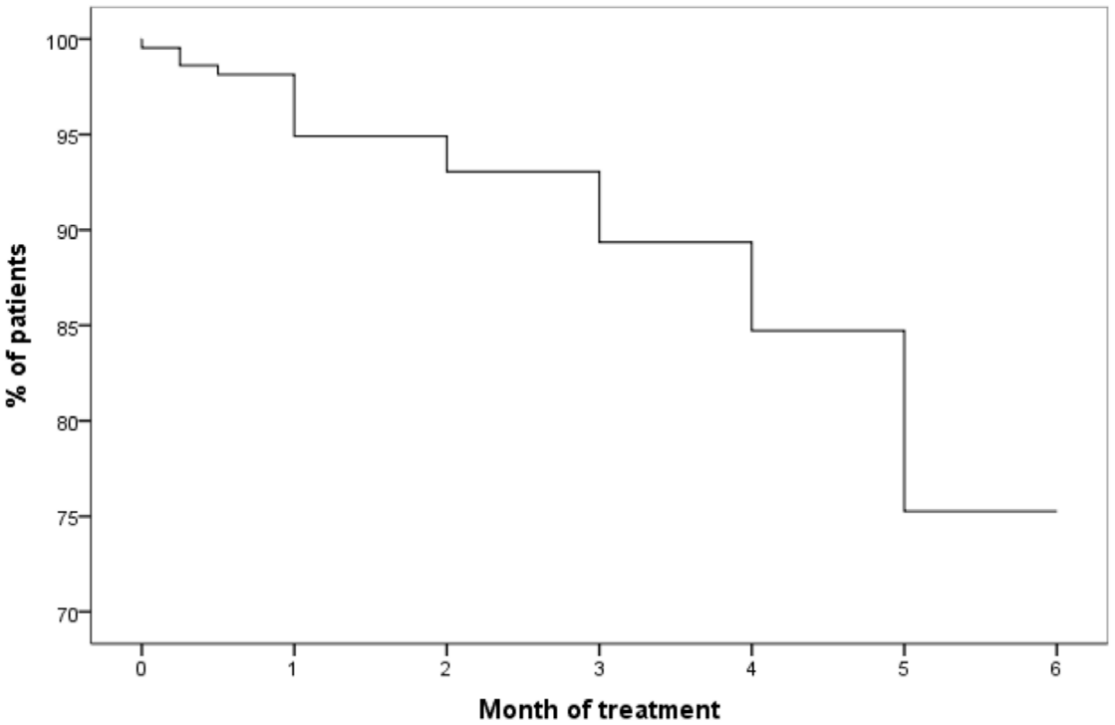
Completion of LTBI treatment. Percentage of patients completing treatment for LTBI by month.

Fifty-three per cent of the 53 patients who did not complete treatment dropped out at some point after three months of treatment, including twenty patients (38%) who had completed five months of treatment (Figure 2). Those who dropped out after completing 5 months treatment took significantly longer to complete a bottle of 30 pills (mean time 38.5 days/bottle, SD 8.2) compared to the group that completed the full 6 months course of treatment (mean time 32.4 days/bottle, SD 5.2) (p<0.001).

### Adverse events

Twelve patients (5%) of 241 patients who were started on isoniazid treatment for LTBI experienced an adverse event thought to be related to isoniazid and were thus not included in the main analysis. Four patients developed a rash, three patients had lethargy and/or mood disorders, two patients had subclinical hepatitis, one patient had symptomatic hepatitis and two patients experienced nausea, vomiting and/or other gastrointestinal symptoms without abnormal liver transaminases. In four patients who experienced an adverse event medication was temporarily ceased and then re-started without change; in three patients the treatment regimen was changed, and in three patients the treatment was ceased completely by the treating physician. Two patients stopped the treatment themselves because of experienced adverse events before consulting a doctor and the treatment remained ceased after review by the treating physician.

## Discussion

In this study, conducted in a TB clinic in Sydney, Australia, a relatively large proportion of people who were started on self-administered isoniazid as treatment for LTBI with monthly drug dispensation through the TB clinic completed treatment. Contrary to the findings of some studies, the majority of people who did not complete treatment dropped out in the second half of the six months treatment course.

The proportion of patients completing treatment in this study was at the higher end of the previously reported range of 19 to 82% for regimens comprising daily isoniazid for 6 to 9 months.[8], [9] Possible reasons for the relatively high completion rate in our study include that treatment for LTBI and all treatment services were provided free of cost to the patients independent of insurance status. Drugs were dispensed directly by the TB clinic nurses and patients were not required to get a prescription filled at another facility. Written information on treatment for LTBI was commonly provided in the patients' primary language. Patients who were overdue for their monthly drug pick up at the TB clinic were contacted up to three times for every missed drug collection or doctors' appointment and encouraged to complete treatment. Free monthly dispensation of isoniazid through TB clinics is generally applied in Australia. Follow-up procedures of patients who do not attend on schedule are likely to vary between different TB clinics. There are no published data on compliance with LTBI treatment from similar other Australian settings. The only published Australian study on compliance with LTBI treatment was in a refugee population in the Northern Territory which found a low completion rate of 44% for those who had been commenced on LTBI treatment mainly due to medication-related side effects and loss to follow up [12].

Although we consider a completion rate of 75% for LTBI treatment with isoniazid over 6 months as quite successful, compared with the previously reported range of completion rates, the overall effectiveness of LTBI treatment as prescribed in this setting is further reduced by the lower efficacy of a 6 months course of treatment compared to a 12 months course of isoniazid (risk reduction of 69% versus 93% respectively [3]). There is a need for a more efficacious LTBI treatment regimen which also facilitates treatment adherence and hence improves overall effectiveness. Recently, a 3 month directly observed once-weekly regimen comprising rifapentine and isoniazid has been compared to a 9 month regimen of daily isoniazid (not directly observed) as treatment for LTBI. The new regimen was associated with a higher treatment completion rate (82%) and equivalent effectiveness for preventing TB [13].

Previous studies have reported contradictory findings for the association between demographic characteristics such as age, sex, place of birth or race and adherence to LTBI treatment [7]. Findings on the effect of time since immigration on completion rates in foreign born people have also been controversial with immigrant status <5 years of residence associated with a lower completion rate in a Spanish study [14], whereas a US study described lower completion rates associated with more years in the US and greater acculturation among immigrants [15]. A positive association with adherence to treatment has been shown for some patient-related factors such as recent close contact with a patient with infectious TB [16], knowledge about TB [17], planning to tell friends/family about evidence of LTBI [18] and marriage [19]. Improved completion rates have also been found for shorter courses of treatment using combinations of drugs or rifampicin alone [7], [20], [21]. Perception of a low risk of progressing to active TB without LTBI treatment [8], the presence of social risk factors [14], [18], alcohol use [19], language barriers [22] and not wanting venepuncture [8] have been associated with decreased completion rates. Our study did not identify any patient characteristics that predicted treatment completion. However, the study may have been relatively underpowered for this outcome.

Adverse events related to isoniazid were associated with lower completion rates. Although adverse events were classified as mild to moderate only and none led to hospitalisation, six out of twelve patients with adverse events did not complete treatment. The high dropout rate of people with adverse events reflects the nature of the decision making process in the decision to commence or continue treatment is based on reconciling the potential advantages and disadvantages of therapy in individual patients. Even mild to moderate adverse effects may change the balance in favour of cessation of treatment. This aspect of the findings in this study is consistent with previous studies [23], [24]. It can be assumed that the reasons for non-completion of treatment in people with adverse events differ from those of people without adverse advents, thus people with adverse events were excluded from the main analysis.

Previous studies have reached varied conclusions about the most common timing for cessation of treatment in those who do not complete therapy. In one US study 54% of those persons who did not complete treatment dropped out before the end of the first month of the treatment course [25]. In another US study of the 36% of patients who did not complete treatment for LTBI with isoniazid 44% received only 2 months of isoniazid therapy, 28% between 3 and 5 months and 28% completed at least 6 months of therapy but did not complete the prescribed nine month course [26]. In contrast, a retrospective study of treatment for LTBI in 19 regions of the USA and Canada found that of the 53% of patients who did not complete a 9 months course of isoniazid, 78% completed 6 months, but failed to complete the final 3 months [27]. Hence, some of the failure to complete treatment in North American studies relates to the final three months of a nine month course, which is longer than the course routinely prescribed in our clinic.

It is possible that the mechanism of drug dispensation, that is monthly drug collection at the TB clinic, influenced the pattern of treatment cessation in our study. Additionally, the lack of a scheduled medical appointment during the final month of treatment may have contributed to the failure to complete the last month of treatment in some patients.

Missing individual doses, which is reflected in the time taken to complete one bottle of 30 tables, may be a predictor of likely treatment completion. Others have found an association between variation in interval between medication doses and taking more than 80% of doses during the first month of treatment and final completion of treatment for LTBI [28], [29]. We also found that the group that dropped out after completing 5 months of treatment took longer to complete a bottle of 30 pills compared to the group that completed the full 6 months course of treatment, which also supports the contention that treatment adherence earlier in the course of treatment could serve as a possible predictor of treatment completion.

The retrospective nature of the study did not allow us to determine the exact reasons for non-completion of treatment for the majority of patients that failed to complete a full course of treatment. A further limitation was that treatment completion was assessed by patients' collection of medication, but actual consumption of isoniazid was not monitored. It is therefore possible that patients returned to pick up another bottle of tablets without having consumed all the tablets from the previous bottle. It would have been ideal to measure detection of metabolites of isoniazid in the urine to evaluate LTBI treatment compliance [30], [31]. However, as this test was not done routinely in the study setting and the study data was collected retrospectively, this information was not available.

In conclusion, in a setting where isoniazid is dispensed monthly by the TB clinic, a relatively high proportion of patients who commence treatment for LTBI complete the six month scheduled course of treatment. The finding that premature cessation was more common in the second half of the scheduled course of treatment implies that interventions should extend over the whole duration of treatment. It is possible that scheduling a doctor's appointment in the last month of treatment may improve the proportion of patients who complete the scheduled course of treatment for LTBI.
