# Correction: Completion of Treatment for Latent Tuberculosis Infection with Monthly Drug Dispensation Directly through the Tuberculosis Clinic

**DOI:** 10.1371/annotation/4498fd07-acae-49bb-abf2-d2e914a249d0

**Published:** 2013-12-09

**Authors:** Claudia C. Dobler, Guy B. Marks

The first sentence under the Results subheading of the Abstract is incorrect. The correct sentence should read: "Of 216 patients who commenced isoniazid treatment for LTBI, 163 (75%) completed six months treatment." 

